# Author response to: Quality of life after open *versus* laparoscopic distal pancreatectomy: long-term results from a randomized clinical trial

**DOI:** 10.1093/bjsopen/zrad069

**Published:** 2023-07-17

**Authors:** Karin Johansen, Anna Lindhoff Larsson, Linda Lundgren, Thomas Gasslander, Claes Hjalmarsson, Per Sandström, Bergthor Björnsson

**Affiliations:** Department of Surgery in Linköping and Department of Biomedical and Clinical Sciences, Linköping University, Linköping, Sweden; Department of Surgery in Linköping and Department of Biomedical and Clinical Sciences, Linköping University, Linköping, Sweden; Department of Surgery in Linköping and Department of Biomedical and Clinical Sciences, Linköping University, Linköping, Sweden; Department of Surgery in Linköping and Department of Biomedical and Clinical Sciences, Linköping University, Linköping, Sweden; Department of Surgery, Hospital of Halland, Halland, Sweden; Department of Surgery in Linköping and Department of Biomedical and Clinical Sciences, Linköping University, Linköping, Sweden; Department of Surgery in Linköping and Department of Biomedical and Clinical Sciences, Linköping University, Linköping, Sweden

Dear Editor

We thank Sharon Barker *et al*. for reading our work and for the important points raised regarding quality of life after minimally invasive and open distal pancreatectomy^1^.

The RCT that included the patients in question was conducted between 2015 and 2019, at which time the exciting prehabilitation concept had not yet gained widespread support. Accordingly, patients were not involved in any specific prehabilitation programme leading up to surgery and were only given general preoperative recommendations of smoking cessation and regular exercise. However, the recommendations were the same regardless of group allocation. The socioeconomic status of the patients, which would have been an important addition to further investigate quality of life results, was unfortunately not included in the study plan and was not available for these analyses. The randomized nature of the study has hopefully helped to divide any socioeconomic differences equally between groups, but as it is a relatively small study size this cannot be taken for granted.

We are fully in agreement with the authors about the need to further investigate the quality of life after minimally invasive distal pancreatectomy in patients with pancreatic cancer, and hopefully the newly closed DIPLOMA (distal pancreatectomy, minimally invasive or open, for malignancy) trial (ISRCTN44897265; www.e-mips.com) will do so. The statement made about minimally invasive treatment already being standard for distal pancreatectomy should be interpreted with caution as this remains to be proven for patients with pancreatic cancer.

## Disclosure

The authors declare no conflict of interest.
